# Characterisation and evaluation of *Metarhizium anisopliae* (Metsch.) Sorokin strains for their temperature tolerance

**DOI:** 10.1080/21501203.2016.1247116

**Published:** 2016-10-27

**Authors:** K. N. P. Chandra Teja, S. J. Rahman

**Affiliations:** a Department of Microbiology, Agri Biotech Foundation, Hyderabad, India; b Department of Environmental Sciences, Jawaharlal Nehru Technological University, Hyderabad, India; c AICRP on Biological Control of Crop Pests and Weeds, PJTSAU, Hyderabad, India

**Keywords:** Entomopathogenic fungi, *Metarhizium anisopliae*, temperature tolerance, radial growth, conidial yield, conidial germination rate, ITS region

## Abstract

Entomopathogenic fungal species from the genera *Beauveria, Metarhizium* and *Lecanicillium* are important components in biological control of insect pests. However, temperature, humidity and UV radiation are among the important abiotic factors, which limit their effective usage. In this study, four local isolates of *Metarhizium* were isolated from different crop rhiospheres of Telangana and Andhra Pradesh states of India, identified and tested for their temperature tolerance in terms of radial growth, conidial yield per 10 mm disc and rate of conidial germination at different incubation temperatures. The results revealed that strains LaMa1 and MaAICRP performed well in terms of radial growth, conidial yield and rate of conidial germination, even at 35°C temperature. The role of such temperature tolerant strains in agriculture is discussed.

## Introduction

Entomopathogenic fungi (EPF) infect insects and are of great importance in biological control of crop pests. EPF are a polyphyletic group comprising approximately 1000 species reported from many taxonomical divisions of the fungal kingdom (Kaya and Vega 2012). Important genera among EPF are *Beauveria, Metarhizium* and *Lecanicillium*. Several species of these genera are in commercial use as Myco-biocontrol agents in agriculture (de Faria and Wraight 2007). These fungi by virtue of their mode of action can directly infect an insect through its cuticle. This property is especially useful in the control of sucking pests. EPF can be isolated either from the insect cadavers during natural epizootics or directly from soil from crop rhizosphere. The two most commonly used methods by researchers for soil isolation are either baiting the soil with an insect host, for example, *Galleria mellonella* (Zimmermann 1986) or using specific selective media (Veen and Ferron 1966; Doberski and Tribe 1980; Beilharz et al. 1982). Since EPF are generally slow growers, other fast growing opportunistic fungi and bacteria dominate while culturing, which is addressed by the selective use of bacterial antibiotics and fungicides.

The efficacy of EPF as biocontrol agents is affected by many biotic and abiotic factors in their environment (Roy et al. 2006), which is one of the major bottlenecks in their usage. Temperature is one such factor affecting the EPF in nature (Ouedraogo et al. 1997). It affects the rate of conidial germination and mycelial development of the fungal pathogens which in turn affect the rate of infection and virulence of these pathogens against the target pests (Nussenbaum et al. 2013; Vidal et al. 1997). Behavioural adaptations of some insect hosts (like locusts) increasing their body temperature to counter fungal pathogens underlines the importance of temperature (Rangel et al. 2010). In contrast, infected drosophila flies displayed behaviour of moving to cooler places to enhance immunity (Hunt et al. 2016). The ability of the certain strains of EPF to grow and sporulate under a wide temperature ranges is very useful in their application as biological control agents particularly in semi-arid climates. This study was undertaken to isolate the entomopathogenic fungal isolates of *Metarhizium* spp. from different agricultural crops of the semi-arid regions of Telangana and Andhra Pradesh states of India and then to study the effect of different incubation temperatures on the radial growth rate, conidial yield and rate of conidial germination of the four isolates to evaluate their temperature tolerance.

## Materials and methods

### Soil sample collection

Eighty-seven rhizospheric soil samples were collected from different crop and forest ecosystems in four districts of Andhra Pradesh and Telangana states, viz. Rangareddy, Warangal, Kurnool and Mahabubnagar districts. Five sub samples of 100 g each were collected from four corners and one at centre of each field. At each site, leaf litter was removed and the soil was collected with a stainless steel borer at a depth of 10–15 cm. The five sub samples were mixed thoroughly in a ziplock bag to make a pooled sample of that field. These samples were brought to the laboratory and stored at 4°C until further processing.

### Isolation of fungi

Thirty grams of sample soil was added to 300 ml of sterile distilled water. The contents were evenly mixed for half an hour and later supernatant was serially diluted up to 10^–6^ dilution. Later, 0.1 ml of sample from 10^–3^, 10^–4^ and 10^–5^ dilutions was spread on to the semi selective medium containing 1% peptone, 2% glucose, 1.8% agar. The antibiotics chloramphenicol (600 mg/l), cycloheximide (50 mg/l) and dodine (100 µl/l) were added to the sterilised medium after cooling it to approximately 60°C. The petri plates were incubated at 25°C for 5–7 days. Colonies morphologically identical to the target fungi were sub-cultured onto fresh Sabraoud’s dextrose agar media amended with 1% yeast extract (SDAY) and identified morphologically using standard taxonomic identification keys (Samson et al. 1988). Purified isolates were stored at 0°C in refrigerator until further use.

### Molecular characterisation of the fungal isolates

The genomic DNA of the fungal isolates was extracted by the chemical lysis method described by Cenis (1992) with few modifications. The concentration of the DNA was quantified and adjusted to 50 ng/µl for its use as template for PCR. The ITS region (ITS1 and 2 and the 5.8S gene) was amplified using the primers ITS1-forward (TCCGTAGGTGAACCTGCGG) and ITS4-reverse (TCCTCCGCTTATTGATATGC) (White et al. 1990). The PCR reaction mixture consisted of 1-µl template DNA of concentration 50 ng, 1 µl of 10 pmol of each primer, 4 µl of 2.5 mM dNTP mix, 2.5 U of Taq DNA polymerase, 5 µl of PCR buffer + MgCl_2_ made up to 50 µl with sterile double distilled water. The PCR reaction involved the following steps of an initial denaturation step at 95°C for 5 min, followed by 36 cycles of (i) denaturation at 94°C for 1 min, (ii) annealing at 56°C for 30 s and (iii) extension at 72°C for 1 min, and a final extension step at 72°C for 10 min. PCR amplified products were separated on a 1% agarose gel stained with ethidium bromide and visualised under UV light along with a 100 bp DNA ladder. The amplicons were purified, and sequenced at Xcelris labs, India using Sanger dideoxynucleotide method and the sequences were compared with the reference sequences in the nucleotide database of NCBI using Blast (https://blast.ncbi.nlm.nih.gov/blast.cgi). The sequences of the isolates were submitted to the NCBI Genbank to obtain the accession numbers. Phylogenetic analysis was done for the isolates using MEGA version 4 (Tamura et al. 2007).

### Effect of temperature on growth

Four isolates of *Metarhizium anispoliae* (Metsch.) Sorokin were used in this study. The isolates were tested for their growth and sporulation in five different temperatures, viz. 25°C, 30°C, 35°C, 40°C, and 45°C.

#### Radial growth

Radial growth of the isolates at different temperatures was determined by fresh inoculation from a 5-mm diameter circular agar disc, cut using a cork borer, from 14-day old culture onto a fresh PDAY medium plate (Bugeme et al. 2008). Five replicate plates were kept for each isolate and for each temperature. The Petri plates were placed in inverted positions incubated at 25°C, 30°C, 35°C, 40°C and 45 ± 0.2°C in respective incubators under dark conditions. The diameter of the colony was measured after 7th, 14th, and 21st day from the day of inoculation of the disc. The measurement was done along the same axis each time.

#### Conidial yield

A 10-mm diameter circular agar disc was cut from the 21-day old culture using a cork borer. Disc was added to 10-ml sterile 0.02% Tween 80 solution and vortexed well for even mixing. The conidial concentration in the supernatant was estimated with the help of a haemocytometer. Conidial yield was expressed as number of conidia per ml

#### Conidial germination

The time taken for the germination of 50% conidia was estimated with a procedure slightly modified from Liu et al. (2003) and Bugeme et al. (2008). The rate of conidial germination of each isolate was assessed by spreading 20 μl of a 10^6^/ml conidial suspension on film of PDAY medium on a glass slide which in turn was kept in polystyrene Petri dishes lined with moist filter paper. The plates were incubated at temperatures 20°C, 25°C, 30°C, 35°C, 40°C and 45 ± 0.2°C in the dark. Germination was recorded under 400× magnifications after 10 h at every 1-h interval until 24 h. A total of 300 conidia were observed in three different fields of observation on the slide. The conidia were considered to be germinated if the germ tube was longer than the width of the conidia. The total numbers of germinated and non-germinated conidia were counted and the time taken for the germination of 50% of the conidia was estimated. Three replicates were kept for each isolate and the procedure was repeated thrice.

### Statistical analysis

The data on radial growth, the conidial concentration and time taken for 50% germination of the conidia were subjected to ANOVA by using Microsoft Excel program. As there was high degree of interaction between temperature and isolates, performance of isolates at different temperatures was separately assessed in one way ANOVA. Means were compared using LSD and tabulated along with their standard deviations. The correlation and regression between the different growth attributes were analysed using Microsoft Excel program.

## Results

### Isolation and identification of fungal strains

Three isolates identified as *Metarhizium* spp. initially using morphological and colony characteristics were isolated by using standard procedures. The source and place origin of the isolates are given in Table 1. KoGn5 was collected from groundnut rhizosphere in Kurnool (15°23′ N, 78°31′ E) district, LaMa1 and PaCo4 from maize and cotton rhizospheres respectively from Mahabubnagar (16°88′ N, 77°55′ E and 16^o^54′ N, 78^o^20′ E) district. One isolate of *Metarhizium* (MaAICRP) originally isolated by the AICRP on Biological Control of Crop Pests and Weeds laboratory at Rajendranagar, Hyderabad, was used in this study. All the isolates were identified as *M. anisopliae* by molecular sequences of the ITS region and homology search in database.

**Table 1.** Source of origin of the isolated *Metarhium anisopliae* strains.

Serial no.	Isolate	Organism	Origin of isolate (rhizosphere soil)	Place	State
1	Ma AICRP	*Metarhizium anisopliae*	Cotton	Rajendranagar, Hyderabad	Telangana
2	KoGn5	*M. anisopliae*	Groundnut	Koilkuntla, Kurnool district	Andhra Pradesh
3	LaMa1	*M. anisopliae*	Maize	Laxmipur, Mahaboobnagar district	Telangana
4	PaCo4	*M. anisopliae*	Cotton	Palem, Mahaboobnagar district	Telangana

### Phylogenetic analysis

**Figure 1.**
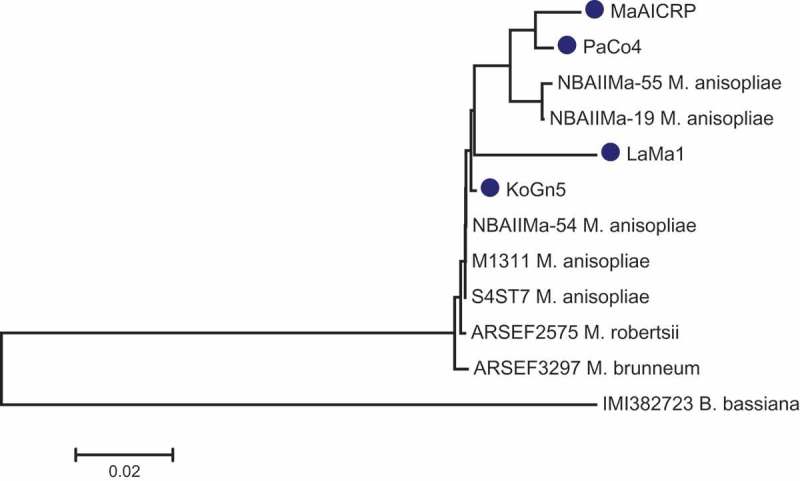
Phylogenetic tree comparing the ITS sequences of *Metarhizium anisopliae* isolates of study (shown in circle) with other species of *Metarhizium* and a *Beauveria bassiana* (IMI3822723) outgroup.

The amplified DNA sequences of the ITS region (Internal Transcribed Spacer 1, 2 and 5.8S subunit) of the four isolates were submitted to NCBI Genbank under the Accession numbers KU647722 (MaAICRP), KT360950 (KoGn5), KT373806 (LaMa1) and KU759902 (PaCo4), respectively. The phylogenetic relationship was derived from comparisons of the ITS gene sequences and a dendrogram was constructed with *B*. *bassiana* as an outgroup. Isolates MaAICRP and PaCo4 are grouped as one cluster and are closely related to NBAIIMa-55 and NBAIIMa-19 of NCBI database. Whereas the isolates LaMa1 and KoGn5 are more distantly related to MaAICRP and PaCo4 (Figure 1).

### Effect of temperature on growth

**Figure 2.**
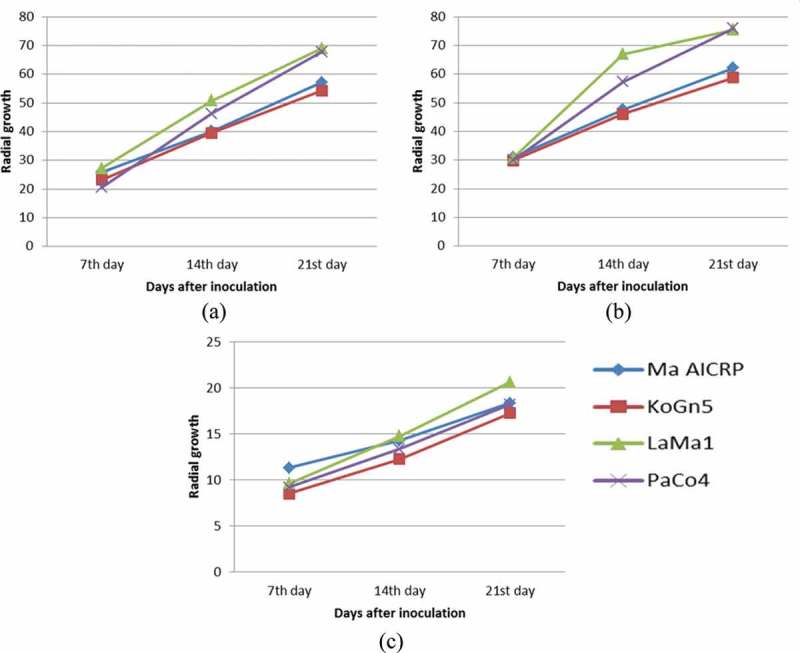
Patterns of growth of the isolates in terms of radial growth (RG) (mm) during the growth period at different temperatures: (a) 25°C (b) 30°C and (c) 35°C.

**Figure 3.**
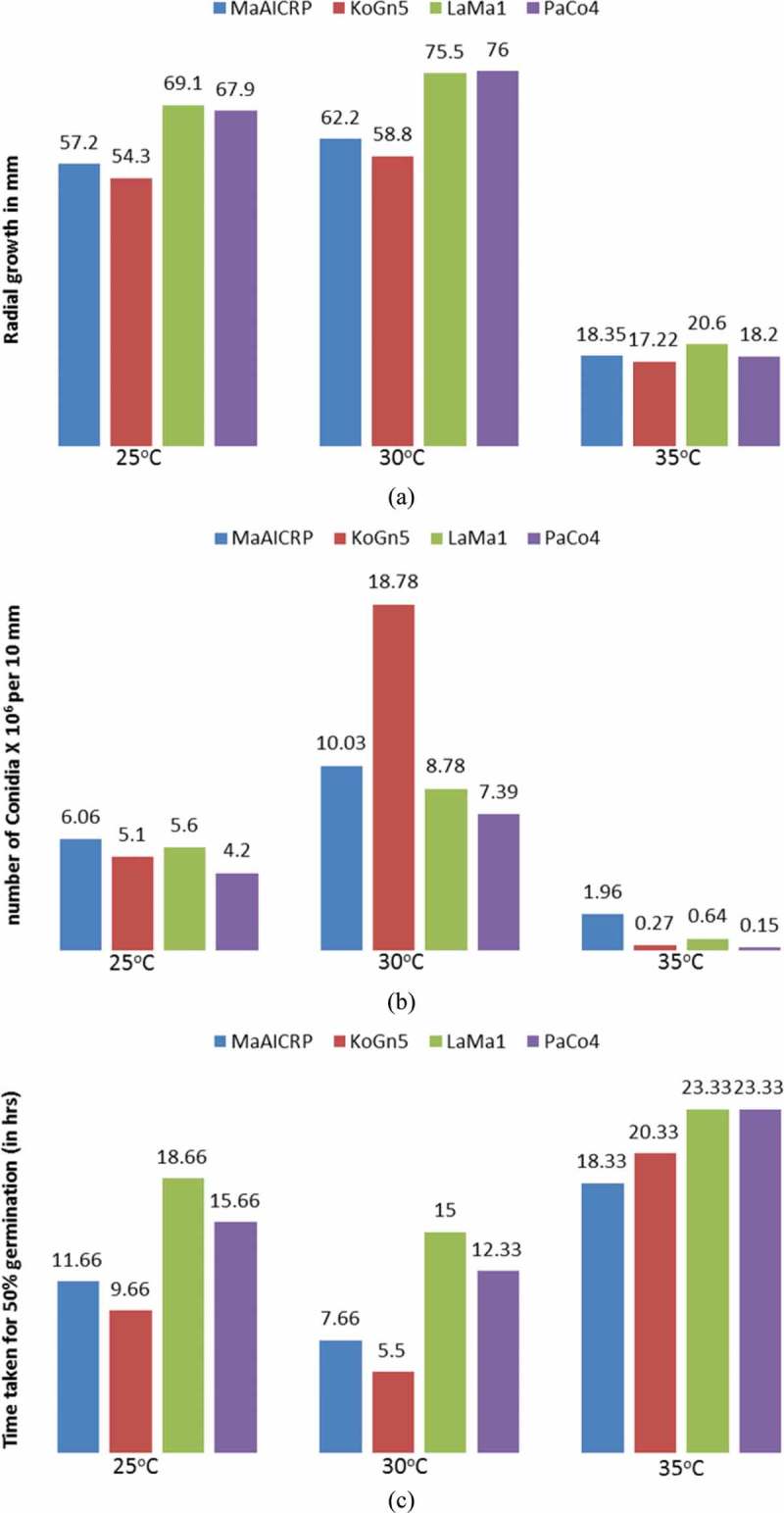
Mean values of the growth attributes of different strains of *M. anisopliae* at different temperatures. (a) Radial growth (RG), (b) conidial yield per 10 mm and (c) time taken for 50% germination.

All the isolates had the maximum radial growth at 30°C followed by those at 25°C (see Figure 3). At 35°C, there was retarded vegetative radial growth and all the isolates failed to grow or form conidia at 40 and 45°C. Significant growth variations were observed not only within the isolates at different temperatures but also among the isolates at a given temperature. While isolates LaMa1 and PaCo4 showed better radial growth than the other two isolates at 25 and 30°C, LaMa1 isolate displayed significantly better performance at 35°C than the rest three isolates. The growth trends of the isolates at 7th, 14th and 21st days at different temperatures (Figure 2) recorded exponential growth from 7th to 21st day at 25°C, but at 30°C, exponential growth was observed only till 14th day and later slowed down. The growth of KoGn5 plateaued after 14th day. Contrary to this, at 35°C, isolates recorded faster growth from 14th to 21st day compared to the first 14 days.

Data on conidial yield per 10mm agar disc did not show a clear trend though all the isolates produced higher yields of conidia at 30°C relative to other two temperatures. KoGn5 isolate at 30°C and MaAICRP at 35°C produced significantly higher conidial yield in comparison with other isolates (see Figure 3). LaMa1 isolate showed higher radial growth at 35°C but had poor conidial yield.

The conidia of the *M. anisopliae* isolates showed germination only at 25°C, 30°C and 35°C temperatures and no germination was observed at 40°C and 45°C even after 72 h of incubation under the experimental setup. Like the other two growth parameters, there was significant variation in the conidial germination rates both among the isolates and incubation temperatures (see Figure 3). All the test isolates germinated significantly faster at the optimum temperature of 30°C with KoGn5 showing the fastest germination (5.50 h). It also showed faster germination rates at all the other tested temperatures except at 35°C wherein MaAICRP isolate showed faster germination (18.33 h). LaMa1 showed the slowest germination among the isolates tested at all the temperatures.

**Figure 4.**
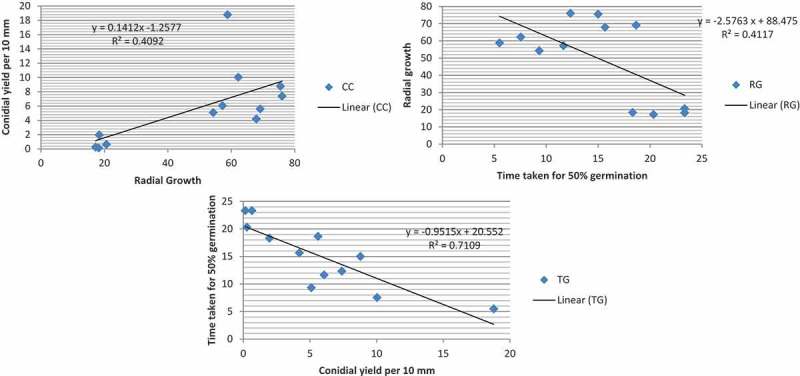
Correlation between the growth parameters: radial growth (RG), conidial yield per 10 mm (CC) and rate of germination (TG50) of the four *Metarhizium* isolates.

The influence of one growth attribute on the other was analysed using correlation and regression methods. It was found that each growth attribute had significant influence on the other (Figure 4). There was significant positive correlation between radial growth and conidial concentration (*R*^2^ = 0.41; *p* = 0.025) while higher concentration of conidia reduced the time for 50% germination (*R*^2^ = 0.714; *p* = 0.0005). In addition, higher radial growth of the isolate was negatively correlated with time for 50% conidial germination (*R*^2^ = 0.4199; *p* = 0.024). Thus faster radial growth may be taken as index of higher biological fitness. Considering all the three parameters, isolate LaMa1 appeared to be the most tolerant to higher temperature of 35°C. Further studies on virulence of these isolates against the target insect *Spodoptera litura* at the test temperatures will provide more support to select the isolate for biocontrol of the pest.

## Discussion

The ITS region DNA sequences of the isolates in this study were compared with other sequences randomly taken from the NCBI database. *B. bassiana* was selected as an outgroup for better estimate of the phylogenetic distance. As expected all the *Metarhizium* spp. clustered distant to the outgroup. The study isolates were evolutionarily more close to other Indian isolates of *M. anisopliae*. Interestingly, the isolates MaAICRP and PaCo4, which clustered with NBAIIMa – 19 and 55 were also geographically closer to the latter. Isolates KoGn5 and LaMa1 were more homologous to NBAIIMa – 54 originating from the northeastern part of India (25°57′ N, 91°88′ E).

The ability of the entomopathogens to adapt to a wide temperature range and survive at high temperatures is a critical factor affecting their success in the field and has been employed as a criterion for the selection of the isolates as biological control agents (Soper and Ward 1981; Maniania et al. 2008). Keeping this in mind, it is very essential to screen the entomopathogenic fungi isolated from different sources for their temperature tolerance. Entomopathogenic fungi in general, have the optimum radial growth at temperatures between 25°C and 30°C (Ekesi et al. 1999; Maniania et al. 2008). However, different strains vary in their tolerance to higher temperatures. Ouedraogo et al. (1997) observed that strains of *M. anisopliae* are better adapted to higher temperatures (35°C) than strains of *M. flavoviride*. Nussenbaum et al. (2013) classified *Metarhizium* strains depending on the optimum temperature for conidial germination as cold active (able to germinate at 5°C), heat active (able to germinate at 37°C) and Meso-thermo active (unable to germinate at either of these temperature).

Dimbi et al. (2004) studied the growth and conidial germination of six *M. anisopliae* isolates and their virulence against African tephritid fruit flies and concluded that the optimum temperature for their growth and conidial germination was 25°C. Thomas and Jenkins (1997) studied the effect of temperature on growth of *M. flavoviride* isolates and found different temperature optima for radial growth and conidial concentration. However, this study did not elaborate to associate higher optimal temperature of the isolate with the collection site or source. Germination of *M. anisopliae* isolates from different geographical locations after brief exposures (2–12 h) to high temperatures (40–50°C) although showed wide variations in the temperature tolerance among the isolates but in general, isolates collected near equator were more tolerant than isolates from high altitudes (Rangel et al. 2005). In this study, all the four *M. anisopliae* isolates showed their maximum growth in terms of all the growth attributes at 30°C temperature. It is imperative since the isolates were collected in tropical areas.

Rangel et al. (2010) studied the upper temperature limit of different species of *Metarhizium* after being exposed to growth inhibiting high temperatures for 10 days. Some of these isolates resumed growth when transferred again to 28°C. Keyser et al. (2014) opined that such a recovery though characterised with delayed growth and reduced infectivity in some isolates is important from biocontrol point of view as the biocontrol agent can tide over the harsh conditions during the cropping season. We also observed that two of the *Metarhizium* isolates, viz. MaAICRP and KoGn5 regained growth after being exposed to 40°C for 14 days and then brought back to 25°C (data not shown).

Although the fungal growth may not have direct influence on its virulence towards the insect pest, there are some evidences that some of the growth attributes affect the performance of fungus on the pest. The infectivity of entomopathogenic fungi increases with the increase in the temperature till the optimum temperature for that particular isolate is reached (Dimbi et al. 2004). Samuels et al. (1989) reported a positive relation between the rate of conidial germination and the virulence of the entomopathogenic fungal strain against the target pest. A study conducted by Ummidi et al. (2013) on *Metarhizium* and *Beauveria* demonstrated that the strains, which are more virulent have significantly faster germination compared to the less virulent ones and suggested that germination rate can be a useful tool to zero down on an efficient strain for biocontrol application. Faria et al. (2015) also noted that the rate of conidial germination is a significant virulence factor and leads to shorter survival times of *Spodoptera frugiperda* larvae.

Milner et al. (1991) while discussing the nutritional requirements for germination of *Metarhizium* strains noted that the rate of germination of conidia is independent of the conidial concentration in the medium. However, our results suggested positive correlation between conidial yield an isolate produced with the rate of germination (*R*^2^ = 0.71) of the isolate. This could be due to some signals or exogenous components from the germinated conidia triggering or accelerating the same in the non-germinated ones. Further study is warranted to establish and confirm this relationship which can ultimately lead to selection of more virulent strains of entomopathogenic fungi.

In summary, this study in order to characterise four local isolates of *M. anisopliae* in terms of their tolerance to high temperatures based on growth, sporulation and germination identified the isolate LaMa1 to be the most temperature tolerant. This information needs to be extended to evaluation of the strains for their virulence against the target pest at growth limiting temperatures.
